# Inulin: A New Adjuvant With Unknown Mode of Action

**DOI:** 10.1016/j.ebiom.2016.11.019

**Published:** 2016-11-15

**Authors:** Mariusz Skwarczynski

**Affiliations:** School of Chemistry and Molecular Biosciences, The University of Queensland, St Lucia, QLD 4072, Australia

Traditionally, vaccines have been produced using whole pathogen cultures, and even today, many vaccines are based on attenuated or killed microorganisms. However, such vaccines, while effective, have significant shortcomings. Not all pathogens can be easily mass produced at the desired developmental stage (*e.g.* malaria sporozoites). Vaccines may induce undesired immune responses, including strong allergic reactions and autoimmunity. Reversion to the virulent form, low-stability, and problems associated with handling of dangerous species during production are other potential drawbacks. Therefore, the use of small antigens instead of whole organisms is becoming more popular in modern vaccine development (Skwarczynski and Toth, 2016). Subunit-based vaccines have a much better safety profile and induce more specific and controlled immune responses. However, they typically lose their danger signals - the microorganism elements recognizable by innate immunity that initiate immune responses. To overcome the loss of “activators of immunity”, immune stimulators (adjuvants) have been introduced to many current vaccine formulations (Barclay and Petrovsky, 2017). Adjuvants are usually recognized by pattern recognition receptors (PRRs), such as Toll-like receptors (TLRs) and C-type lectin receptors displayed by antigen presenting cells (APCs). However, many potent adjuvants (*e.g.* complete Freund's adjuvant) suffer from significant toxicity. Only a few adjuvants have been used in human vaccines, and all of them (except alum) are approved for one particular vaccine only. The limited choice of safe adjuvants has generated strong interest in the development of new immune stimulating molecules and/or vaccine delivery systems, as exemplified by inulin, reported by Ishii and coworkers in this issue of *EBioMedicine* (Hayashi et al., 2017).

Inulin (Advax™, β-d-[2 → 1] poly(fructo-furanosyl) α-d-glucose) is a plant-derived carbohydrate. It has no immunological activity in soluble form; however, once it is formulated into delta inulin microparticles, its adjuvanting activity is widely acknowledged (Petrovsky and Cooper, 2015). Inulin was applied to enhance vaccine efficacy against influenza, hepatitis B, West Nile virus, Japanese encephalitis, human immunodeficiency virus, SARS, and anthrax, amongst others. The carbohydrate was effective and safe in both experimental animals, as well as in humans. Yet, despite the large number of studies performed, its mechanism of action is still unclear. This prompted Ishii and coworkers to investigate the potential mode of action of inulin microparticles. At first, they investigated its adjuvanting capacity when administered together with antigens already bearing danger signals. It has been reported that influenza split vaccine (SV) elicits Th2, while whole virion influenza vaccine (WV) triggers a Th1 response. When antigens were delivered with inulin, immune responses were significantly increased and the Th1/Th2 direction remained unchanged. And when inulin was delivered with “danger signal free” ovalbumin as an antigen, nothing happened. No antibody production against ovalbumin was detected. Moreover, inulin on its own was not able to stimulate dendritic cell (DC) maturation *in vitro*. Maturation of DCs is the crucial step before adaptive immunity can be activated. Just taking into account these observations, it might be assumed that inulin acts as a delivery system, possibly by preventive antigen degradation or through improved delivery to APCs, but without its own immune stimulating abilities.

Is inulin really just a delivery platform for vaccines, without adjuvanting properties? The answer is no. In contrast to *in vitro* testing, *in vivo* DC maturation experiments showed that inulin acted as an adjuvant and enhanced the expression of maturation markers on these cells. The reason for such unique behavior of inulin is yet to be determined. In addition, Ishii and coworkers demonstrated that DCs and phagocytic macrophages, as well as tumor necrosis factor (TNF)-α played a crucial role in the adjuvanting ability of inulin. It is of note that the ability of inulin to enhance adaptive immune responses when injected a day earlier than an antigen was reported previously (Saade et al., 2013). Thus, inulin acts as an unusual adjuvant, as it did not force the direction of immune response (Th1 *vs* Th2), as typical adjuvants do. For example, commercially-approved alum is a well-known Th-2 pathway stimulator, while CpG-ODN triggers Th1 response (Azmi et al., 2014).

The article in focus brings us closer to understanding the way inulin interacts with the immune system; however, further investigation is still required to disclose its mechanism, or mechanisms, of action. As soon as we understand how inulin interacts with the immune system, we can start manipulating inulin. Once the mechanism(s) of action is known, the carbohydrate molecule can be modified to improve its adjuvanting capacity, antigen can be chemically incorporated into inulin, and so on. Alternatively, the research order could be inverted, and modifications could be made as a means for deciphering the mechanism of action of inulin. Regardless of which approach proves most effective, an understanding of inulin's mechanism of action will be crucial not only in improving its efficacy, but also in fully establishing its safety profile, especially in immune impaired/dysregulated individuals, such as the elderly.

## Disclosure

The author declared no conflicts of interest.
